# Neural correlates of confusability in recognition of morphologically complex Korean words

**DOI:** 10.1371/journal.pone.0249111

**Published:** 2021-04-15

**Authors:** Jeahong Kim, JeYoung Jung, Kichun Nam

**Affiliations:** 1 Department of Psychology, Korea University, Seoul, Republic of Korea; 2 School of Psychology, University of Nottingham, Nottingham, United Kingdom; University of Valencia, SPAIN

## Abstract

When people confuse and reject a non-word that is created by switching two adjacent letters from an actual word, is called the transposition confusability effect (TCE). The TCE is known to occur at the very early stages of visual word recognition with such unit exchange as letters or syllables, but little is known about the brain mechanisms of TCE. In this study, we examined the neural correlates of TCE and the effect of a morpheme boundary placement on TCE. We manipulated the placement of a morpheme boundary by exchanging places of two syllables embedded in Korean morphologically complex words made up of lexical morpheme and grammatical morpheme. In the two experimental conditions, the transposition syllable within-boundary condition (TSW) involved exchanging two syllables within the same morpheme, whereas the across-boundary condition (TSA) involved the exchange of syllables across the stem and grammatical morpheme boundary. During fMRI, participants performed the lexical decision task. Behavioral results revealed that the TCE was found in TSW condition, and the morpheme boundary, which is manipulated in TSA, modulated the TCE. In the fMRI results, TCE induced activation in the left inferior parietal lobe (IPL) and intraparietal sulcus (IPS). The IPS activation was specific to a TCE and its strength of activation was associated with task performance. Furthermore, two functional networks were involved in the TCE: the central executive network and the dorsal attention network. Morpheme boundary modulation suppressed the TCE by recruiting the prefrontal and temporal regions, which are the key regions involved in semantic processing. Our findings propose the role of the dorsal visual pathway in syllable position processing and that its interaction with other higher cognitive systems is modulated by the morphological boundary in the early phases of visual word recognition.

## Introduction

Words are essential elements of our modern society. We live in the world surrounded by words; everywhere and every day we see and use words (e.g., newspapers, Twitter, shop and street names, traffic signs, etc.). Thus, it is important to understand how our brain processes words. Psychologists have discovered an interesting phenomenon in visual word recognition related to the perceptual position processing: The transposition confusability effect (TCE), which occurs when two adjacent letters within a word are switched to create a new string of letters that form a new non-word. Due to this, we confuse a transposed letter non-word (*jugde*–*judge*; transposition condition) with the original word, but not a word formed by the replacement of two letters (*jubqe*–*judge*; replacement condition) in lexical decision tasks (LDT) [1–5] and form-priming tasks [6–10]. Since the first published papers that compared transposition letter condition with replacement letter controls [8, 9], differences between two conditions (transposed letter non-word vs replacement of two letters) in reaction time and error rate (longer reaction time and greater error rate in lexical decision task and shorter reaction time in form-priming task for the transposed condition compared to the replacement condition) were considered as TCE in behavioral studies.

In visual word recognition, it is important to recognize the letters in a given word (bottom-up processing) as well as the position of the letters that constitute a given stimulus (top-down processing). TCE is known to occur in the very early stages of visual word processing and is caused by the top-down processing of human contextual memory, which involves the position of the letter string in visual word recognition [4, 11]. Thus, TCE provides information on a fundamental aspect of visual word recognition, which is whether the letter is processed position-specifically or not. However, recent evidence suggests that the TCE can occur on the basis of purely perceptual noise [12]. In addition, numerous studies reported the TCE for other strings, such as digits and symbols and novel letters [13–15]. The magnitude of TCE was greater for words (or letters) than other stimuli, suggesting orthographic or lexical involvement in the TCE. Thus, the TCE has been reported mainly in language with its orthographic perceptual chunks (i.e., letters or syllables), which are linear and concatenated.

As the transposition effect is related to the position perceptual processing, several visual word recognition models assume that this phenomenon is occurred at the early stage of visual word recognition. Open bigram models [16] suggest that the processing of two letters chunking. For example, when a reader reads a word ‘stand’, certain steps are taken to recognize every possible bigram such as ‘st’, ‘sa’, ‘sn’, ‘sd’, ‘ta’, ‘tn’, ‘td’, ‘an’, ‘ad’, and ‘nd’. If there is only one drop out of the bigram ‘an’ in a transposed pseudoword, ‘stnad’ compared to a replaced nonword, ‘stmed’, then TCE would occur for the transposed pseudoword. Spatial coding models [17–19] suggest that the first letter would have the greatest activation and the activation would be decreased for latter letters. The activation level of the transposed pseudoword ‘stnad’ is more similar to that of the real word, ‘stand’ than the activation level of the replaced nonword, ‘stmed’, as they shared same 5 letters with 3 positions. The overlap model [20] suggests that each letter has a different degree of activation with differential influence on neighboring letters. This model assumes if the sum of all letter activation in a transposed pseudoword has similar to that in a read word ‘stand’, then it would lead to TCE. Therefore, the transposed pseudoword ‘stnad’ causes the transposition confusability because it has more overlapping activation distribution with the real word ‘stand’ than the replaced nonword ‘stind’. Although the TCE have been widely investigated in psycholinguistics, the neural mechanism underpinning this phenomenon remains unclear.

While the letter transposition effect has been found in Western languages (e.g., English, Spanish, etc.), Korean studies have failed to find the letter transposition effect [21, 22]. However, recent studies reported the transposition effect in Korean syllables—a CV or CVC sequence in Korean (Korean letters are formed into a syllabic block-shaped cluster [23]). Hangul, the Korean writing system, is composed of a syllable and the syllable has a morphological component in Hangul, but not in Western languages. Lee and his colleagues investigated the syllable transposition effect and found slower rejection times in the case of a transposition of the middle two syllables of four-syllable words (i.e., 해***욕수***장 for 해수욕장 ‘beach’) than for matched control replaced nonwords (e.g., 해***욕주***장), using the nonword rejection paradigm [24]. Our recent study [25] also found the syllable transposition effect in Korean four-syllable inflected nouns, in which the transposition of the middle two syllables showed a significantly longer rejection time and higher error rate than matched replaced nonwords. These results indicated that the syllable could played a more major perceptual role than letter in Korean visual word recognition. Syllable satisfies the characteristics of being linear which is one of the primary requirements for transposition effect and also includes morphological components in Korean writing system. This syllable transposition effect seems robust phenomenon in other syllabic units based languages such as Chinese character [26, 27] and Japanese kana [28, 29].

In contrast, the syllable transposition effect has not been strongly suggested in western languages. Perea and Carreiras [4] manipulated two letters forming a syllable transposition (syllable transposition condition, PRIVEMARA-primavera, the Spanish for spring) and two letters not forming syllable transposition (bigram transposed condition, PRIMERAVA-primavera). They reported that the magnitudes of transposition effect were similar for both conditions, indicating that the effect was not syllabic in Spanish. In an English study, Crepaldi and his colleagues [30] found the morpheme transposition effect (e.g., honeymoon to moonhoney), but not the syllable only transposition effect. Taken together, the TCE at the syllable level is a robust phenomenon in East-Asian languages compared to the Western languages.

Transposition confusability effect also provides the temporal information of the other lexical units (i.e., morpheme). Morpheme is known as the smallest lexical unit that contains meaningful information. Researchers have debaed upon whether morphological processing is bound with orthographic information at the earlier stages of visual word recognition or it is bound with semantic information at a later stages of visual word recognition. Studies that support the morpho-orthographic processing have reported the morpheme boundary effect on the TCE—slower reaction times in the case of transposition within a morpheme (e.g., *violinist* in Spanish violinista, vio***il***nista—within the morpheme boundaries) than for transposition across a morpheme boundary (viol***in***sta—across a morpheme boundary) [31–35]. As the TCE is considered to occur at a very early stage of visual word recognition [36, 37], researchers argue that the morpheme boundary effect modulating the TCE is attributable to early orthographic processing. In contrast, other studies have reported no differences between across-morpheme and within-morpheme conditions, supporting the claim of morpho-semantic processing [38–41]. Recently, Dunabeitia and his colleagues demonstrated that faster readers showed a decreased TCE modulated by the morpheme boundary, but slower readers did not show such effect [33]. They suggest that the morpheme boundary effect on the TCE depends on the individual reading proficiency. Although many studies have investigated the effect of morpheme boundary modulating the TCE, there is no consensus on whether this effect is associated with orthographic or semantic processing.

Although many studies have examined the behavioral aspects of the transposition effect, research on how our spatial system in the brain processes this effect is still lacking. There have been only a few brain imaging studies examining the TCE. Carreiras et al. [42] conducted an fMRI study to investigate the TCE at the visual perceptual stage using the priming paradigm. They used letters (KBTG), numbers (8267), and symbols (%?+<) for both the transposition condition (K**TB**G–KBTG, 8**62**7–8267, %**+?**<–%?+<) and the replacement condition (K**LP**G–KBTG, 8**39**7–8267, %**&)**<–&?+<). The behavioral results demonstrated TCE for all transposition conditions, especially in the letter condition. They found greater activation in the bilateral inferior parietal lobe (IPL), bilateral superior parietal gyrus (SPL), and right angular gyrus in the transposition condition than the replacement condition. Additionally, they reported greater activation in the left IPL and SPL for the letter condition than the other two conditions, suggesting that TCE was present in visual processing irrespective of the type of stimuli, which suggests that visual word processing was more sensitive to confusability than the other types of visual stimuli. The IPL and SPL were involved in the general confusability effects, while the left hemisphere seemed to be more involved in the language-related confusability effect. Lin et al. [27] investigated the brain activation of TCE at the word recognition level with an unprimed LDT. They used Chinese words of two characters and compared transposable nonwords with regular nonwords, and reported the activation of the bilateral IPL for TCE. They suggested that the left IPL was associated with the semantically-related transposition confusability, whereas the right IPL was related to Chinese-character processing. These studies demonstrated that IPL plays a crucial role in the TCE during visual word processing.

However, the understanding of the neural mechanism underpinning the TCE is still lacking, especially for its modulatory effect (i.e., effects involving morpheme boundaries). Here, we conducted a rapid-event-related functional magnetic resonance imaging (fMRI) study to examine the neural correlates of TCE and morpheme boundary effect using morphological complex Korean words. Participants were asked to perform an unprimed LDT with morphologically inflected Korean nouns. There were three conditions: the transposed across morpheme condition (TSA), the transposed within morpheme (TSW) condition, and the replacement condition as a control. Based on previous findings, we expected the involvement of the IPL in the TCE. As previous behavioral studies have reported the role of morpheme boundary acting as a cue to eliminate transposition confusability effect, we also investigated the neural mechanisms of the morpheme boundary effect in suppressing or eliminating the TCE when it involves crossing morpheme boundaries (TSA). Furthermore, we explored the functional connectivity of the TCE-related brain regions for an advanced understanding of their roles in visual word recognition at a network level.

## Materials and methods

### Participants

Twenty-eight right-handed healthy volunteers participated in the experiment. Three participants’ fMRI data were excluded due to excessive head movements (over 1.2 mm in one or more directions). The reported results reflect data from the remaining 25 participants (16 females) ranging in age from 20 to 26 years (mean = 22.64, *SD* = 1.89 years), all native Korean speakers. All participants had normal or corrected to normal visual acuity and no psychiatric or neurological records. Participants provided written consent before the experiment. This study was approved by the Ethical Committee of Korea University.

### Stimulus

A lexical decision paradigm was employed in this experiment. Four-syllable *eojeol*, a unique type of Korean morphologically complex word, were selected from the Korean Word Database [43] to meet the requirement of three experimental conditions: 20 transposed-across-morpheme nonwords (TSA, e.g., 친**에구**게 – 친구에게 ‘to (a) friend’; the first two syllables comprise the lexical morpheme and the last two syllables the grammatical morpheme), 20 transposed-within-morpheme nonwords (TSW, e.g.,목**리소**가 – 목소리가 ‘voice is’; the first three syllables comprise the lexical morpheme and the last syllable the grammatical morpheme), 20 nonwords which involved the replacement of one of the middle-two-syllables (RS, e.g., 며**키느**가 – 며느리가), and two filler conditions, which included 40 regular words and 20 baseline masks (e.g., “####”), were employed. Korean letters are formed into syllabic block-shaped clusters [21], and Korean words are written by concatenating written syllables whose boundaries are clearly divided, which reflects that Korean is an agglutinative language that has morphologically complex words as its basic form. This characteristic enabled us to investigate the clear differentiation of the morphological boundary between the experimental conditions. In this study, we used inflected Korean words composed of a noun and a postposition, which are lexical and grammatical morphemes, respectively. The last syllable of the lexical morpheme and the first syllable of the grammatical morpheme were transposed in the across-morpheme condition (구 in the lexical morpheme 친구 and 에 in the grammatical morpheme 에게), while the second and last syllables of the lexical morpheme were transposed in the within-morpheme condition (소 and 리 in the lexical morpheme 목소리). A total of 120 trials were included in the experiment (S1 Table). Three lexical factors—whole word frequency, stem frequency, and first syllable token frequency—were statistically matched (Table 1), as they have been reported to be significant in Korean-noun morphologically complex word recognition [44].

**Table 1:** Descriptive lexical variables by experimental condition.

	TSA (per mil)		TSW (per mil)		RS (per mil)		
	Log mean (*SD*)	Range	Log mean (*SD*)	Range	Log mean (*SD*)	Range	Statistics
Whole word frequency	1.246 (.351)	3–72	1.336 (283)	6–76	1.323 (.435)	5–181	n.s.(*F* = .363, *p* = .697)
Stem frequency	2.077 (.389)	11–452	2.043 (.273)	22–339	2.052 (.557)	6–693	n.s.(*F* = .034, *p* = .967)
1^st^ syllable token frequency	3.374 (.386)	343–8507	3.542 (.547)	341–33534	3.462 (.591)	69–14874	n.s.(*F* = .538, *p* = .587)

All frequencies were transformed to per mil values and statistical analyses were conducted on log-transformed values.

### Experimental design

**Fig 1:**
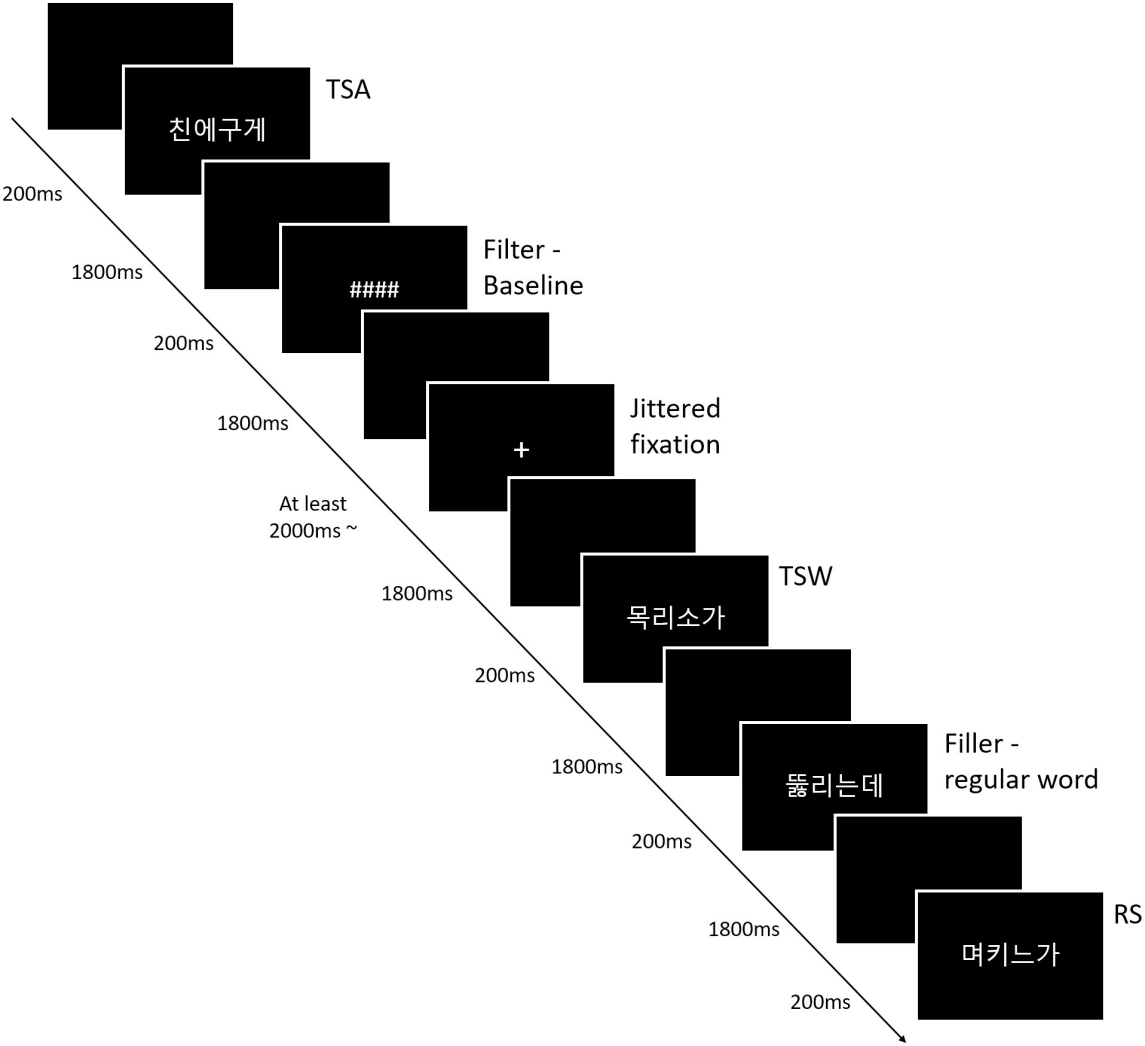
Experimental design.

A rapid-event-related fMRI design was employed in the experiment. All stimuli were presented in the center of a black background screen in white 34-point font. In each trial, the target stimulus was presented for 200ms followed by a blank for 1800ms. A fixation cross (“+”) with a jittered interval of at least 2s was used as a null condition inserted randomly between task conditions (Fig 1). The order of test trials and the length of jittered stimuli were optimized using Optseq software (http://surfer.nmr.mgh.harvard.edu/optseq/). All participants reached accuracies of over 80% during the practice session.

### Image acquisition

A Simens Magnetom Trio 3T MRI scanner at the Korea University Brain Imaging Center, Seoul, South Korea, was used for this study. A T2*-weighted-gradient Echo Planar Imaging (EPI) sequence was used to acquire Blood Oxygenated Level Dependent (BOLD) fMRI images with the following parameters: TR = 2000ms; TE = 20ms; Flip Angle = 90°; Field of View = 240 mm; slice thickness = 3 mm, no gap 42 slices; matrix size = 80 × 80; and voxel size = 3 mm × 3 mm × 3 mm. High-resolution anatomical images (1 mm × 1 mm × 1 mm) were acquired for each participant, with a T1-weighted, 3D MPRAGE (Magnetization-Prepared Rapid Gradient-Echo) sequence (TR = 1900ms;TE = 2.52ms; Flip Angle = 90°; Field of View = 256 mm; matrix size = 256 × 256).

### Behavioral analysis

The one-way ANOVA and Bonferroni corrected post-hoc were conducted for task accuracy and reaction time. The analyses were done with correct rejection of TSW, TSA, and RS in the experiment. Only correct trials were used for reaction time analysis. SPSS 24 was used for the analyses.

### fMRI data analysis

SPM12 (http://www.fil.ion.ucl.ac.uk/spm/software/spm12/) was used for functional image analysis. The first three functional volumes were discarded to reduce the transition effects of hemodynamic responses. The remaining images were first realigned for motion correction, slice timing, co-registration, and segmentation, and then spatially normalized to a standard MNI (Montreal Neurological Institute) template. Before the analysis, the images for every participant were smoothed with an isotropic Gaussian kernel of 6mm FWHM.

At the individual level, the data were modeled using general linear modeling (GLM). Conditions were modeled in an event-related design, and the BOLD signal was convolved with a standard hemodynamic response function (HRF). Movement parameters estimated from the realignment procedure were entered as regressors. Only correct responses were included in the analysis. For each participant, individual contrast images between the experimental conditions (TSA / TSW / RS) and baseline mask conditions were created. The following six contrasts were estimated at this stage (TSA > RS, TSW > RS, TSA > mask, TSW > mask, TSA > TSW, TSW > TSA).

In the random effect analysis, the contrast images derived from the individual level analysis were analyzed using a one-sample *t*-test. The contrast TSW > RS was related to the transposition confusability effect, while the contrast TSA > RS was associated with the suppression or disappearance of the transposition confusability effect. The contrasts of transposed conditions > mask (TSA > mask and TSW > mask) were related to the transposed word processing taking into account the language process. The comparison between TSA and TSW conditions was the main point of interest in this study. The resulting whole-brain maps were thresholded at p < 0.001 at the voxel level with False Discovery Rate (FDR)-corrected cluster threshold of p < 0.05, ks > 30. The findings and labels for activated brain regions are reported according to the MNI atlas.

A region of interest (ROI) analysis was carried out to estimate the level of activation using the MarsBar toolbox [45]. Based on the GLM results (the contrast of TSA > RS and TSW > RS), four ROIs with a 5-mm radius sphere were created: the left dorsomedial prefrontal cortex (mPFC, MNI [−3, 56, 8]), left middle temporal gyrus (MTG, MNI [−54, −28, −10]), left inferior parietal lobe (IPL, MNI [−48, −55, 47]), and left intraparietal sulcus (IPS, MNI [−24, −64, 35]). To evaluate the task effect, a one-sample *t*-test was performed for all ROIs and a paired *t*-test was performed to compare the morpheme boundary effect in different conditions (*p* < 0.05).

### Functional connectivity analysis

The CONN-fMRI toolbox (http://web.mit.edu/swg/software.html) was used to estimate functional connectivity (FC) from a seed region to the whole brain. Four ROIs from the GLM results (i.e., mPFC, MTG, IPL, and IPS) were used as a seed region. FC is computed from the temporal correlation between brain activity in a seed region and the whole brain using a GLM approach. We created four additional ROIs based on previous studies [42, 46] to test whether the morpheme boundary effect on the TCE is processed with orthographic or semantic information. It includes the regions associated with orthographical processing—the left posterior fusiform gyrus (pFFG, MNI [−48, −60, −17]), left posterior middle occipital gyrus (pMOG, MNI [−40, −77, −1]), and left anterior fusiform gyrus (aFFG, MNI [−34, −38, −16])—and the semantically-related area, the left posterior middle temporal gyrus (pMTG, MNI [−54, −41, −2]). The ROI-to-ROI FC was computed between the IPL and these ROIs. Pre-processed images were entered in the toolbox. Head movements were entered as regressors at the individual-level analysis. In order to remove motion, physiological, and other artifactual effects, denoising was conducted using CompCor [47]. Data were detrended, despiked, and filtered with a bandpass filter (0.01 < *f* < 2) to decrease the effects of low-frequency drift and the influence of potential outlier scans. In the first-level analysis, a seed-to-voxel analysis was performed for each subject per condition. The corresponding residual BOLD time course from the ROI was extracted and Pearson’s correlation coefficients between the extracted time course and the time course of all other voxels were computed. The maps were *z*-score normalized and one-sample *t*-tests were used to find areas of significant positive correlations with the seed region (*p*_FDR-corrected_ < 0.05, *k*s >30, *p*_uncorrected_ < 0.001 at a voxel level).

## Results

### Behavioral results

**Fig 2:**
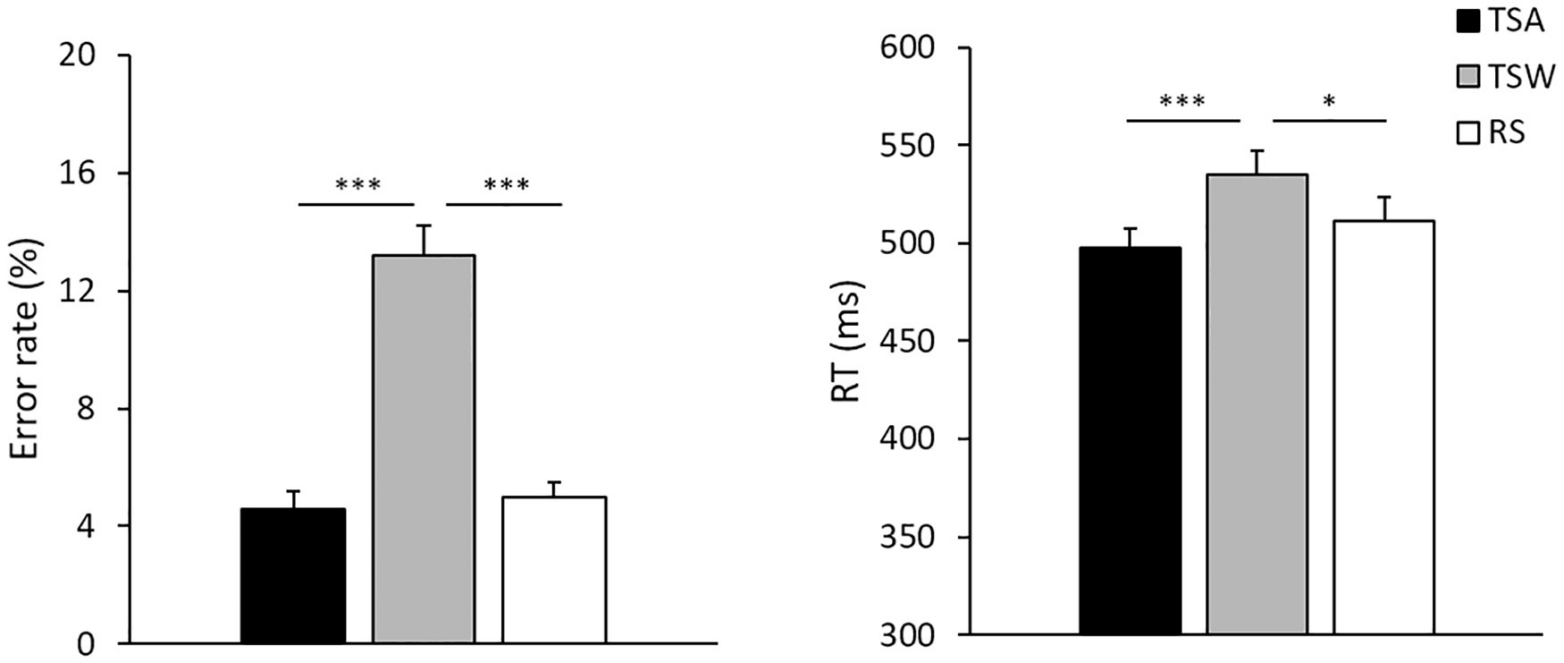
Behavioral results of reaction time (RT) and error rate for TSA, TSW, and RS conditions. Note: *p < .05, **p < .01, ***p < .001.

Reaction times (RTs) of correct trials and error rates were collected as the participants performed the lexical decision task in the scanner. Mean RTs of the TSA, TSW, and RS nonword conditions were 498.83ms (*SD*: 95.82), 535.72ms (*SD*: 124.1), and 511.4ms (*SD*: 126.59), respectively (Fig 2). For reaction time, one way repeated measure ANOVA showed main effect [*F*(2,48) = 6.057, *p* = .005, *η*^2^ = .483] and post-hoc (Bonferroni corrected p) showed the TSW was marginally slower than the RS [*p* = .070], and significantly slower than TSA [*p* = .006]. However, the TSA showed no significant difference compared to the RS [*p* = .870]. The mean error rates for the TSA, TSW, and RS nonword conditions were 4.6% (*SD*: 6.1%), 13.2% (*SD*: 10.9%), and 5% (*SD*: 5.4%), respectively (Fig 2). As with RTs, the error rate showed main effect [*F*(2,48) = 12.264, *p* < .001, *η*^2^ = .338] and post-hoc showed the error rate of the TSW was significantly greater than RS [*p* = .003] and the TSA [*p* = .003] and no difference between the TSA and the RS [*p* = 1.00]. The results showed that the transposition confusability existed in the TSW condition, but not the TSA condition.

### GLM results

**Fig 3:**
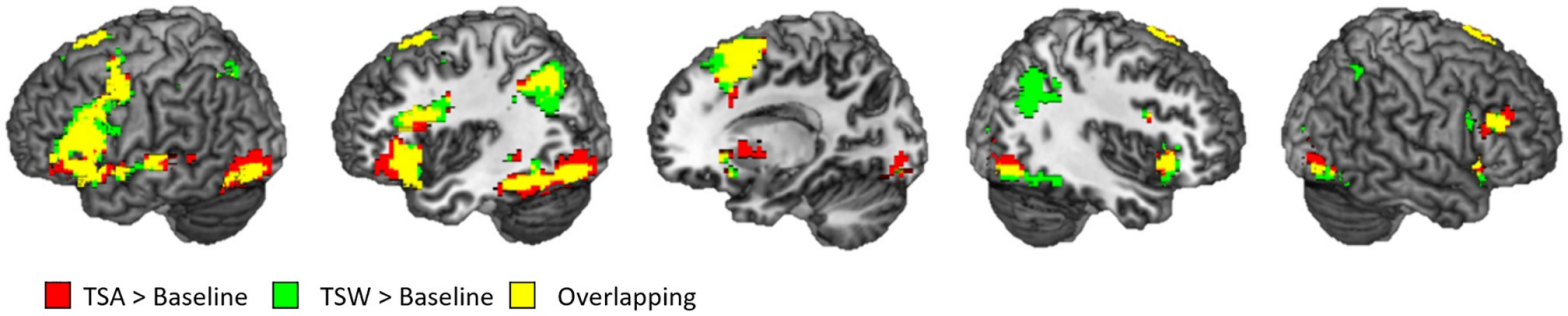
The results of whole brain analysis. Red color indicates TSA > Baseline, green color indicates TSW > Baseline, and yellow color indicates overlapping brain areas. *p* < .05 FDR-corrected at a cluster level, *p* < .001, *K*s > 30 at a voxel level.

The whole-brain activation areas of the experimental conditions are reported in Table 2 and Fig 3. A wide range of language-related brain areas were activated for both TSA and TSW compared to the baseline, including the inferior frontal cortex, middle temporal gyrus (MTG), fusiform area, inferior parietal lobe (IPL), supplementary motor area (SMA), putamen, and superior occipital gyrus (Fig 3). TSW showed additional activation in the right supramarginal gyrus.

**Table 2:** Brain areas for each type of transposable nonword condition, relative to the baseline condition.

Contrast	Regions	Cluster Size	MNI Coordinate (x, y, z)	Peak t value
**TSA > baseline**	Inferior Orbito Frontal gyrus	966	−48 23 23	9.69
	Fusiform gyrus	324	−33 −88 −10	9.21
	Superior Occipital gyrus	444	15 −88 2	8.68
	Putamen	110	30 23 −7	7.19
	Supplementary Motor Area	291	0 20 47	6.70
	Inferior Triangular Frontal gyrus	62	45 8 23	6.64
	Caudate	48	−18 11 2	6.63
	Middle Temporal gyrus	148	−48 −37 2	6.50
	Inferior Parietal lobe	102	−24 −61 44	5.70
	Inferior Triangular Frontal gyrus	39	45 38 26	5.11
**TSW > baseline**	Inferior Orbito Frontal gyrus	1022	−39 11 29	7.96
	Supplementary Motor Area	436	−3 14 62	7.32
	Inferior Triangular Frontal gyrus	97	48 14 23	7.27
	Inferior Parietal lobe	310	−24 −61 32	7.15
	Middle Temporal gyrus	106	−48 −43 2	6.95
	Superior Occipital gyrus	158	15 −82 −13	6.77
	Fusiform gyrus	74	42 −76 −10	6.77
	Fusiform gyrus	151	−45 −46 −16	6.76
	Inferior Triangular Frontal gyrus	38	48 32 23	6.04
	Supramarginal gyrus	174	30 −67 29	5.79
	Putamen	116	39 26 −4	5.49
	Cerebellum	35	−6 −79 −28	5.32
	Inferior Parietal gyrus	30	−39 −46 44	4.70

The regions reported for contrasts with baseline condition were *p* < .05 FDR-corrected at a cluster level, *p* < .001, *K*s > 30 at a voxel level.

**Fig 4:**
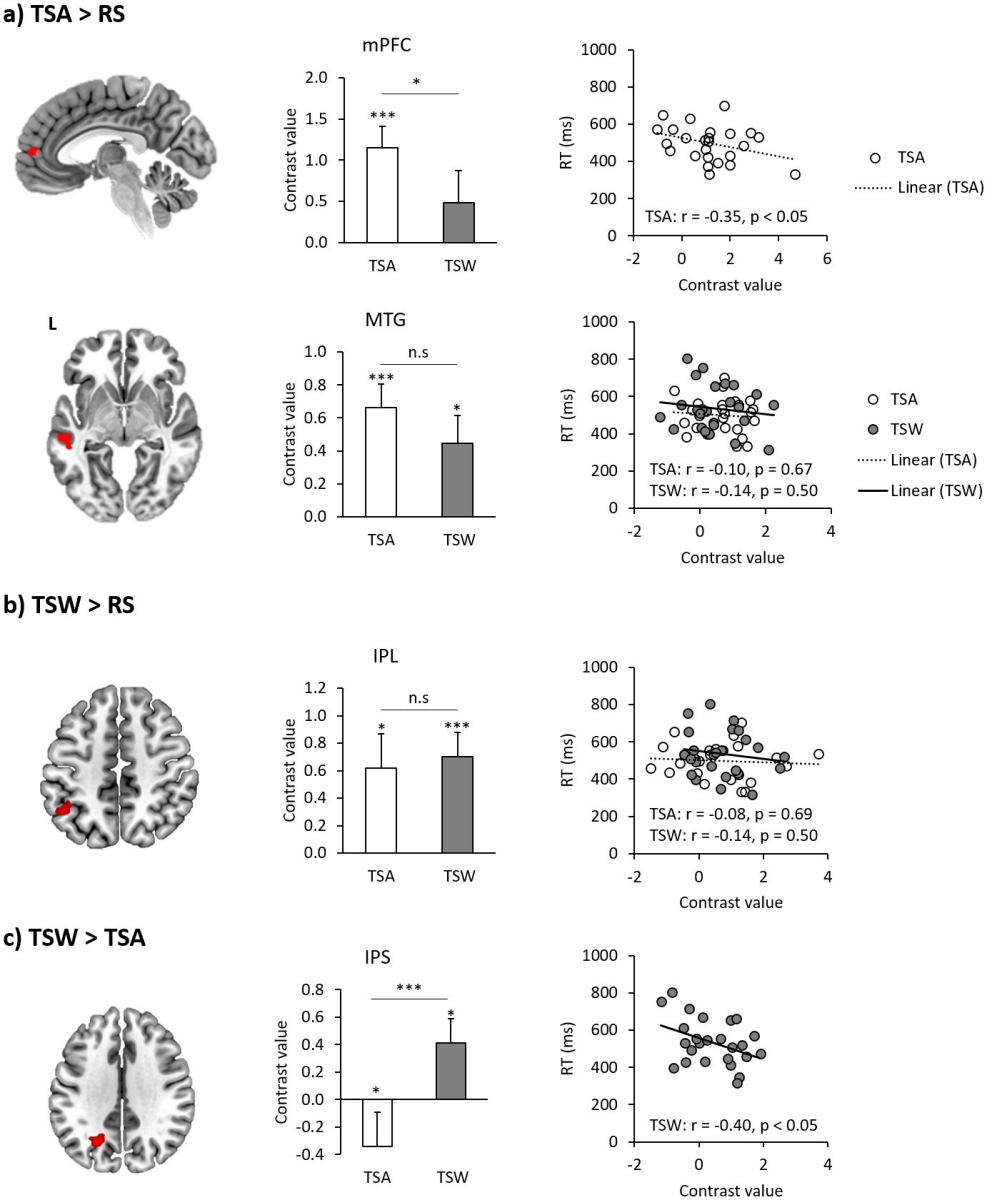
fMRI analysis of the TSA and TSW contrasts with RS. (a) The whole brain activation map of TSA > RS (up: mPFC, down: Left mMTG); (b) whole brain activation of TSW > RS (left IPL); (c) whole brain activation of TSW > TSA (left IPS). Left column shows whole brain activation map, middle column gives ROI results, and right column presents a correlation plot between beta value and behavioral data (reaction time). ** *p* < .01.

Compared to the RS condition, TSA condition showed significant activation in the left MTG and the rostral part of the mPFC (Fig 4a). TSW condition relative to RS condition revealed activation in the left IPL (Fig 4b). We found significantly greater activation in the left intraparietal sulcus (IPS) for TSW than for TSA (Fig 4c). No voxel survived for the TSA minus TSW contrast. The results are summarized in Table 3.

**Table 3:** Brain areas for each type of transposable nonword condition, relative to the replacement condition.

**TSA > RS**	Middle Temporal Gyrus	42	−54 −28 −10	3.84
	Medial Prefrontal Cortex	38	−3 56 8	3.47
**TSW > RS**	Inferior Parietal Lobe	37	−48 −55 47	4.87
**TSW > TSA**	Intra-Parietal Sulcus	51	−24 −64 35	5.94

The regions reported for contrasts with baseline condition were *p* < .05 FDR-corrected at a cluster level, *p* < .001, *K*s > 30 at a voxel level.

We conducted ROI analysis to investigate the effect of transposition confusability across and within-morpheme (TSA and TSW) during visual word processing. The mPFC showed a significant involvement of the TSA [*t*(24) = 4.306, *p* < .001] and greater activation for TSA than TSW [*t*(24) = 1.975, *p* = .029] (Fig 4a). The MTG was significantly activated for both TSA [*t*(24) = 4.603, *p* < 0.001] and TSW [*t*(24) = 2.626, *p* = .015], and there was no difference between the conditions (Fig 4b). The IPL also showed significant activation for both TSA [*t*(24) = 2.498, *p* = .020] and TSW [*t*(24) = 4.357, *p* < .001]. The IPS was more activated in TSW than in TSA [*t*(24) = −5.284, *p* < .001], revealing significant deactivation in TSA [*t*(24) = −2.391, *p* = .025] and activation in TSW [*t*(24) = 2.357, *p* = .027] (Fig 4c). In order to explore the relationship between the level of activation in the ROIs and task performance, correlation analyses were conducted. We found that the activation of the mPFC was negatively correlated with the RT for TSA [*r* = .345, *p* = .046] (Fig 4a). The level of activity in the left IPS had a significant negative correlation with TSW [*r* = .405, *p* = .022] (Fig 4c). The other regions did not show any significant correlations with either TSA or TSW.

To confirm our findings, we also conducted an additional ROI analysis with a priori ROI, the left IPL (-54, -51, 36) from the previous work by Lin et al. [17]. We replicated our finding showing the left IPL’s involvement for TSW and TSA conditions. The left IPL was significantly activated for both TSA>RS and TSW>RS contrasts (S1 Fig).

### FC results

**Fig 5:**
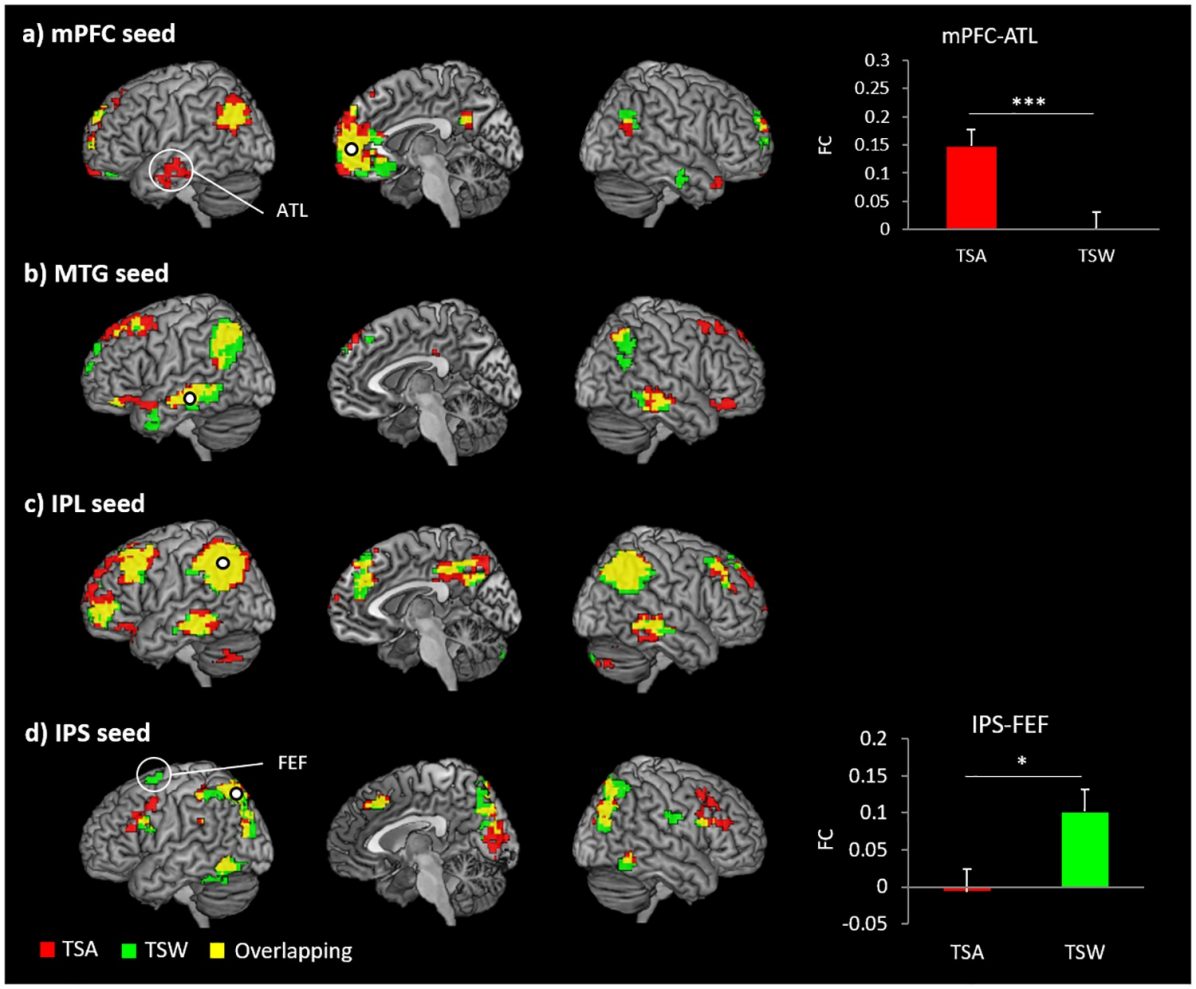
Functional connectivity results. Red indicates TSA condition, green indicates TSW condition, and yellow indicates overlapping. *** p < 0.001, * p < 0.05.

The mPFC seed showed positive connections with areas previously associated with the default mode network (DMN), including the bilateral AG, precuneus, posterior cingulate cortex (PCC), and superior frontal gyrus (SFG) for both TSA and TSW (Fig 5a). During TSA, the mPFC was significantly coupled with the bilateral anterior temporal lobe (ATL). The mPFC-ATL connectivity was significantly increased in the TSA condition compared to the TSW condition (*t*(24) = 4.059, *p* < .001) (Fig 5a). We found that the mPFC was positively correlated with the right MTG during TSW. The MTG seed was significantly coupled with the IFG, SFG, IPL, AG, and the right MTG regardless of task conditions, which are part of the central executive network as well as the semantic network (Fig 5b). The IPL seed showed a similar pattern of FC with the MTG, suggesting that they are part of the same functional network. Positive coupling was found between the IPL and bilateral IFG, DLPFC, ACC, precuneus/PCC, IPL, and MTG across TSA and TSW (Fig 5c). The IPS showed positive connections with regions associated with the dorsal attention network (DAN), including the bilateral IFG, MFG, IPL, SPL, superior/middle occipital gyrus, inferior temporal gyrus, fusiform gyrus, precentral gyrus, and SMA regardless of task conditions (Fig 5d). In particular, the IPS was positively coupled with the FEF during the TSW. This IPS-FEF connectivity was stronger in the TSW condition relative to the TSA condition (*t*(24) = 2.757, *p* = .011) (Fig 5d). The results of the seed-based FC analysis are shown in Fig 5 and S2 Table.

**Fig 6:**
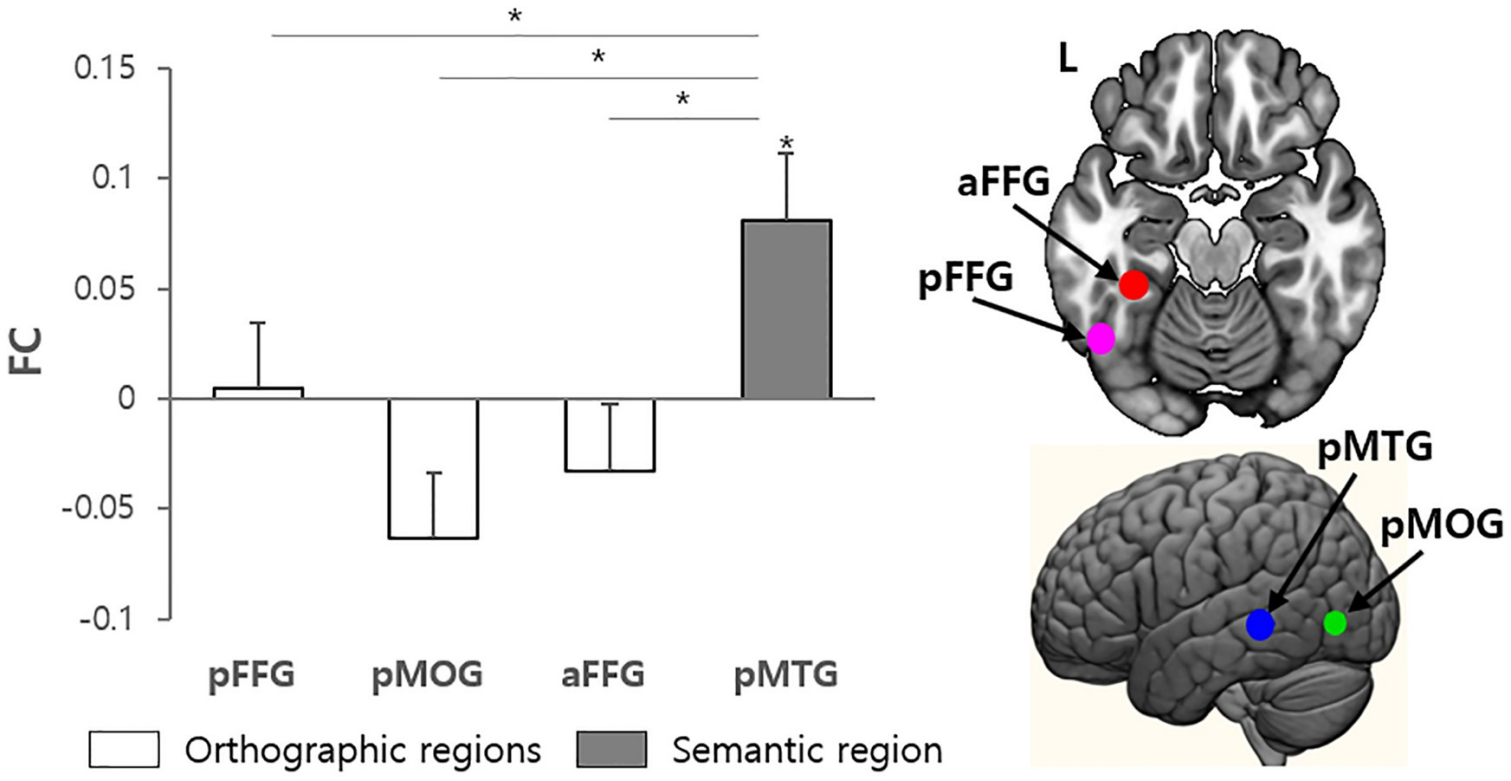
The results of the FC between the IPL and orthographic regions (pFFG, pMOG, and aFFG) and semantic area (pMTG) during the TSW. * *p* < .05.

To investigate the neural mechanism of the morpheme boundary effect, the ROI-to-ROI FCs were calculated for the IPL with the orthographic regions (i.e., aFFG, pFFG, and aMOG) and semantic area (pMTG) in TSW (Fig 6). The IPL was strongly coupled with the pMTG [*t*(24) = 2.269, *p* = .033] in TSW. The increased FC between the IPL and pMTG was greater than the other FCs (IPL-pFFG [*t*(24) = 2.144, *p* = .042], IPL-pMOG [*t*(24) = 2.505, *p* = .019], and IPL-aFFG [*t*(24) = 2.153, *p* = .042]).

## Discussion

The transposition confusability effect occurs when we confuse a non-word which is created by switching two adjacent letters within the word during reading. This effect provides the meaningful information related to the positional processing of the orthographic chunks (letters in Indo-European languages and syllables in East Asian languages) at the early stages of visual word recognition, suggesting that the stored position of orthographic chunks can influence the processing of the visually given input word. On the other hand, morpheme boundary effect has been studied in the transposition effect paradigm to investigate whether or not the morphological processing occurs at the earlier stages of visual word recognition. The elimination of the transposition confusability effect is thought to be related to the morpheme boundary acting as a cue in suppressing the transposition confusability effect. Here, we investigated this interesting phenomenon with two key objectives: (a) to map the neural correlates of TCE using morphologically complex words and (b) to explore the effect of morpheme boundary on the TCE at regional and brain network level. Our results showed that TCE induced activation in the left IPL and IPS. The IPS activation was specific to TCE and its degree of activation was associated with task performance. Furthermore, two functional networks were involved in the TCE, the central executive network (CEN) and the dorsal attention network (DAN). The morpheme boundary modulation suppressed the TCE by recruiting prefrontal and temporal regions—the key regions in the default mode network (DMN) and the semantic network (SN). Our findings suggest that the dorsal visual pathway plays a critical role in the position processing of sub-lexical units (i.e., syllable) and its interaction with other higher cognitive systems is modulated by the morpheme boundary in the early phases of visual word recognition.

Vidyasagar [48, 49] has provided a neural model of early spatial selection in visual word recognition and reading based on the dorsal and ventral visual pathways [50]. The ventral pathway extending into the left inferior temporal cortex plays a major role in object identification, while the dorsal pathway projecting to the parietal regions mediates spatial processing such as motion, depth, and object location [51]. With neural evidence [52, 53], this model suggests that the dorsal pathway plays a role in the early selection of features in spatial processing by identifying and selecting relevant regions in space to be passed on the ventral stream or back to early visual areas for more detailed processing [49]. Recent investigations of the TCE demonstrated the involvement of the left IPL, showing increased activation during the transposition condition [27, 42]. In particular, Carreiras et al. [42] reported greater activation in the left IPL for letter strings than for symbol and digit strings. Expanding their findings into the word level, Lin et al. [27] demonstrated the IPL activation with the transposed nonwords compared to the regular words in Chinese. These findings support the involvement of the dorsal pathway in early visual word recognition. Similarly, we found the activation in the IPL and IPS in the transposition conditions with morphologically complex Korean Eojeols. These findings are consistent with those results reported in previous studies which employed letters and Chinese characters [27, 42], supporting the role of IPL in the TCE. Furthermore, we demonstrated that the IPS activity was specific to TSW. Importantly, participants with stronger activation in the IPS performed the task faster. Different from English letters and Chinese characters, Korean Eojeols consist of the lexical morpheme and the grammatical morpheme making it morphologically complex [54]. Such morphologically complex composition of Korean Eojeol might have contributed to the IPS activation found in the TSW compared to the TSA condition. The IPS is thought to play a role in visual attention [55] and visuospatial working memory [56]. Specifically, it has been reported that there was an increase in the activity when the task was more demanding [57, 58]. Thus, the complexicity of Eojeol and the disruption of its processing caused by transposed position might be associated with the increased IPS activity. Along with the activation in the ventral visual pathway, our findings highlight the involvement of the IPL in syllable positioning processing, especially the crucial role of the IPS in TCE. They support the involvement of the dorsal visual pathway and its interaction with the ventral visual pathway in the earlier stages of visual word processing [48, 49]. The involvement of the posterior region is in line with the previous transposition ERP studies [59–62].

Our FC analysis showed that two distinctive functional networks were involved in the transposition conditions with respect to the morpheme boundary modulation. The IPL seed revealed the CEN, consisting of the bilateral dorsal prefrontal cortex (DLPFC), anterior cingulate/pre-supplementary motor area, posterior MTG, and IPL [63, 64], while the IPS seed showed the DAN, including the frontal eye field (FEF) and IPS [64, 65]. The CEN is involved in executive processing across domains, increasing activation for more demanding conditions and tasks [66]. The DAN is associated with top-down attention by biasing sensory stimuli [65]. It should be noted that there was significant coupling between the IPS and FEF only in the TSW condition, and that the lateral occipital cortex (LOC) was connected with the DAN in both transposition conditions. These findings indicate that the task-active domain general network was required during the transposition condition, and TCE condition further recruited top-down attentional processing. Previous studies reported the involvement of visuospatial and visual attentional processing of an unfamiliar visual format [67–69] and in reading visually unfamiliar letter strings [70–73]. For successful visual word recognition in TCE, visual attention is required to shift attention from one letter to another [48]. Neuropsychological evidence also supports this, showing that impairment of the dorsal pathway was associated with poor reading skills [74, 75]. As the functional networks involved in the TCE does not fully depend on the full coding of positional chunks processing [61], our findings provide evidence of active top-down attentional processing in TCE when rejecting the confusable pseudoword as a nonword in visual word recognition.

We showed that the morpheme boundary successfully modulated the TCE. As expected, behavioral data showed TCE in the TSW condition and a reduction of the TCE in the TSA condition. These findings are consistent with the previously reported morpheme boundary modulation of the TCE [31–35]. fMRI data revealed increased activation in the mPFC and left MTG when the morpheme boundary fell between syllables. Specifically, the mPFC showed greater activation in the TSA than the TSW and participants with stronger activity in the mPFC performed better in the task (faster RT). Theories of the mPFC have suggested its role in adaptive decision making and memory [76]. In particular, neuropsychological evidence indicates that the mPFC plays a critical role in both short-term and long-term memory across a broader range of tasks [77, 78]. The mPFC is a part of the DMN, which is deactivated during goal-directed tasks [79], but a recent study has reported that semantic processing modulates the DMN, especially the mPFC [80], and others have also shown that mPFC activation is related to semantic representation [81, 82]. Here, we demonstrated that the mPFC was connected to the left ATL during the TSA. The ATL is a trans-modal hub in semantic memory [83]. Recent work has demonstrated significant coupling between the ATL and mPFC when the semantic demand was increased in a given task [84]. These findings suggest the involvement of semantic processing when the morpheme boundary is positioned between syllables. In addition, we observed the left MTG activation during TSA. As a key region of the language network, the MTG is associated with semantic processing [83]. In particular, the MTG plays a crucial role in semantic control along with the IFG, which guides the semantic memory system to select a particular concept or generate an appropriate behavior in a given task or context [85–87]. In our data, we observed connectivity between the MTG and ventral IFG in the TSA condition. Thus, our results indicate that morpheme boundary modulation allows the access of semantic interference in the transposed syllables by recruiting the semantic networks, leading to the reduction of TCE in visual word recognition. Our results provide evidence that the morpheme boundary can act as a cue for suppressing the confusability effect in the early stage of visual word processing, and that this may be attributed to the action recruited by the semantic system.

Our findings have important implications in relation to the long debate in visual word recognition over the argument on whether visual word recognition gains access to morphological information before lexical identification, or rather upon lexical identification. We examined the FC between the IPL and regions associated with orthographic and semantic processing in order to elucidate the morpheme boundary effect on the TCE. Our results provide evidence that the structural violation in morpheme boundary does in fact act as a cue in eliminating TCE and may be attributed to semantic processing, showing increased FC between the IPL and pMTG in the TSW condition. Also, we found the involvement of mPFC and increased FC with ATL and MTG during TSW. These findings demonstrate that semantically-related brain regions are involved in morpheme boundary processing, suggesting the role of morpho-semantic processing in visual word recognition [88]. Lin et al. [27] investigated the TCE using Chinese multi-character words and found increased activation in the IPL along with semantically related brain regions such as the ATL, mPFC, and angular gyrus. They suggest that the involvement of the IPL may support the recognition of Chinese multi-character words by accessing and/or integrating semantic information during Chinese word reading. Together, these findings imply that the IPL plays a crucial role in TCE, processing the positional information as well as semantic information via the interaction with semantic-related regions at the level of sub-lexical units (e.g., Korean syllables and Chinese characters).

Our results seem to be incompatible with the temporal flow of visual word processing such as the feedforward processing from orthographic to semantic information. However, recent studies using electroencephalography and magnetoencephalography have reported the early processing of semantic information around 200ms of onset word presentation [89–94]. In particular, the ATL and pMTG, the key regions of semantic cognition, showed increased activity/synchronization around 200–250ms [95, 96]. A study employing an electrocorticogram with 10 patients who had undergone subdural electrode implantation demonstrated increased activity in the ATL starting from 250ms [97]. Thus, the involvement of semantic regions found in our fMRI data might indicate the higher-level modulation of the frontal-temporal-parietal to occipital cortices in the early visual word processing. To test this possibility, future studies using methods with better temporal resolution will be needed.

The current study has several limitations. First, Korean Eojeol comprehension might be possible with the help of some other processes other than the word recognition itself. Because the Eojeol has its phrasal characteristics, it consists of lexical and grammatical morphemes that corresponds to the English phrase stracture (e.g. 친구에게 –to friend). Thus, Korean Eojeol processing might be different from word processing. However, our fMRI results showed that Eojeol processing was supported by the word recognition system including the IFG and ventra-temporal regions such as visual word form area [47, 98] (Fig 1). Also, the purpose of this study was to investigate the neural mechanism of the TCE and the morpheme boundary effect on TCE. Previous studies have reported emergence of TCE when using Korean four-syllable words [24] and Korean Eojeols [25]. By confirming the TCE in Korean Eojeol at behavioural level, we examined its neural correlates in this study. Our findings replicated previous studies employing characters letters, numbers, and symbols, showing the invovlvment of the IPL for the TCE [27, 42]. The TCE in Korean Eojeols was also supported by the dorsal visual pathway. Second, the length of stem in the TSA and TSW conditions was different. We carefully created the four- syllable stimuli accounting for the morpheme boundary as well as three lexical factors: word frequency, stem frequency, and the1st syllable token frequency. These factors have been reported to influence Korean Eojeol recognition [44]. When trying to match the stem length for each stimulus, it required more than four syllables, hence resulting in longer stimuli (e.g., five-syllable long Eojeols). However, for five-syllable Eojeols, it was not possible to take into account the three other lexical factors. It should be noted that the RS stimuli have both 2-syllable and 3-syllable stems to match with the TSA and TSW conditions (see the S1 Table). Third, our ROI analysis might have caused the double dipping issue [99]. To avoid this, we took the IPL (MNI -54, -51, 36) from Lin et al. [27] as a ROI to confirm our results (see S1 Fig). The results replicated our initial findings, which showed increased IPL activation in both TSW and TSA conditions. There was no difference between two conditions. However, our study was the first to investigate the morpheme boundary effect on the TCE. Thus, there was no priori ROIs for the regions associated with the TSA condition. Therefore, future studies should consider using stimuli with controlled length of the stem to confirm and elucidate our current findings. Fourth, it should be noted that the FC results showed only the pattern of connectivity during the task conditions. The direct comparison of TSW and TSA did not show any survived brain regions. The ROI-to-ROI FC analysis demonstrated differntial connectivity between the seeds and regions according to the task conditions which means our results showed diffential patterns of connectivity between two conditions, not the specific activation of each condition. Finally, our findings did not provide information of the temporal course of TCE in visual word recognition. To examine it, future ERP/MEG studies will be needed to clarify the time course of the TCE.

## Conclusion

In visual word recognition, the boundary between bottom-up and top-down processing has been debated for a long time, with research focusing on whether or not the visual input feeds into the lexical level in a hierarchical manner or whether higher-level linguistic information such as morphological structure exerts a top-down influence on orthographic processing at earlier processing stages [100]. Here, by employing the TCE, we demonstrated the involvement of the left parietal lobe during visual word processing, and the role of the morphological boundary in reconciling the TCE by recruiting the frontal and temporal cortices. Furthermore, seed-based FC analysis revealed that TCE was involved in the DAN as top-down attentional processing, while the semantic system was associated with the decrease of TCE modulated by morpheme boundary. Our findings suggest that letter identification and position processing are not only tasks in which the ventral pathway is involved but are also associated with interactions of the dorsal visual pathway with other higher cognitive systems in the early phases of visual word recognition.

## Supporting information

S1 Fig Results of the ROI from Lin et al. 2016. (DOCX)

S1 Table The list of stimuli. (DOCX)

S2 Table Whole brain connectivity table for each seed region. (DOCX)
