# Supplementary material for: Neural correlates of confusability in recognition of morphologically complex Korean words
Source: PLoS One. 2021 Apr 15;16(4):e0249111. doi: 10.1371/journal.pone.0249111 (PMC8049294; doi:10.1371/journal.pone.0249111)
Supplement: S1 Fig — (DOCX) [file pone.0249111.s001.docx]

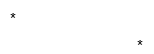

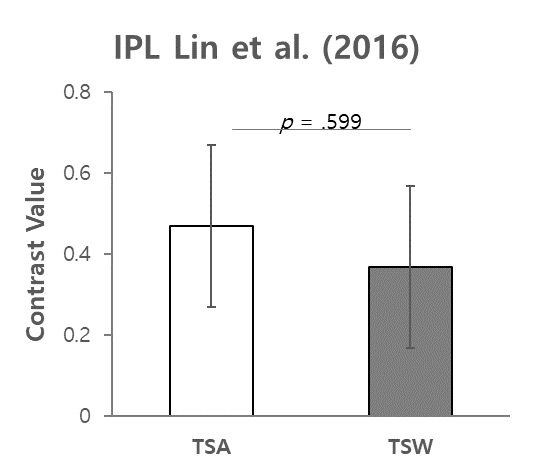


**S1 Fig**. Results of the ROI from Lin et al. 2016

We conducted ROI analysis of left IPL (-54 -51 36) activation from Lin and his colleagues [17] to avoid double dipping problem in fMRI ROI analysis and find replicable the left IPL’s involvement for TSW and TSA condition. Results showed significant activation for TSA>RS [*t*(24) = 2.30, *p* = .015, mean = 0.467] and TSW>RS [*t*(24) = 1.90, *p* = .035, mean = 0.368] contrast and showed no significant differences [*t*(24) = .533, *p* = .599].
