# Supplementary material for: Neural correlates of confusability in recognition of morphologically complex Korean words
Source: PLoS One. 2021 Apr 15;16(4):e0249111. doi: 10.1371/journal.pone.0249111 (PMC8049294; doi:10.1371/journal.pone.0249111)
Supplement: S1 Table — (DOCX) [file pone.0249111.s002.docx]

**S1 Table. The list of stimuli**

| Condition | Item | | Morpheme | |
| --- | --- | --- | --- | --- |
|  | Transposed | Original | Lexical | Functional |
| Transposed Syllable  Across Morpheme  (TSA) | 겨에울는 | 겨울에는 | Winter | Topic |
|  | 근으간로 | 근간으로 | Foundation | Allative |
|  | 기으분로 | 기분으로 | Mood | Allative |
|  | 내부년터 | 내년부터 | Next year | Source |
|  | 독에자게 | 독자에게 | Reader | Beneficial |
|  | 바으탕로 | 바탕으로 | Background | Allative |
|  | 배으경로 | 배경으로 | Scenery | Allative |
|  | 병에원서 | 병원에서 | Hospital | Locative |
|  | 부에엌서 | 부엌에서 | Kitchen | Locative |
|  | 선에거서 | 선거에서 | Election | Locative |
|  | 전으문로 | 전문으로 | Professional | Allative |
|  | 조으건로 | 조건으로 | Condition | Allative |
|  | 직으선로 | 직선으로 | Straight line | Allative |
|  | 진으정로 | 진정으로 | True | Allative |
|  | 최에근는 | 최근에는 | Recent years | Topic |
|  | 측에면서 | 측면에서 | Side | From |
|  | 친에구게 | 친구에게 | Friend | Beneficial |
|  | 표으정로 | 표정으로 | Expression | Allative |
|  | 현에장서 | 현장에서 | Spot | From |
|  | 회에의서 | 회의에서 | Meeting | From |
| Transposed Syllable  Within Morpheme  (TSW) | 거말짓을 | 거짓말을 | Lie | Accusative |
|  | 공체동의 | 공동체의 | Community | Possessive |
|  | 그자림가 | 그림자가 | Shadow | Nominative |
|  | 목리소가 | 목소리가 | Voice | Nominative |
|  | 문점제을 | 문제점을 | Problem | Accusative |
|  | 민당주의 | 민주당의 | Democratic | Possessive |
|  | 분기위를 | 분위기를 | Atmosphere | Accusative |
|  | 비기행를 | 비행기를 | Airplane | Accusative |
|  | 사실무을 | 사무실을 | Office | Accusative |
|  | 쓰기레를 | 쓰레기를 | Rubbish | Accusative |
|  | 어움려이 | 어려움이 | Difficulty | Nominative |
|  | 에지너를 | 에너지를 | Energy | Accusative |
|  | 이크라에 | 이라크에 | Iraq | Locative |
|  | 자차동를 | 자동차를 | Car | Accusative |
|  | 재부판는 | 재판부는 | Judiciary | Topic |
|  | 컴터퓨를 | 컴퓨터를 | Computer | Accusative |
|  | 프스랑는 | 프랑스는 | France | Topic |
|  | 한디마로 | 한마디로 | Short | allative |
|  | 할니머가 | 할머니가 | Grandma | Nominative |
|  | 화실장을 | 화장실을 | Toilet | Accusative |
| Replaced Syllable  (RS) | 국헤민게 | 국민에게 | People | Beneficial |
|  | 기애지를 | 기지개를 | Stretch | Accusative |
|  | 단틸백을 | 단백질을 | Protein | Accusative |
|  | 당가사인 | 당사자인 | Party | Descriptive |
|  | 며피느가 | 며느리가 | Daughter-in-law | Nominative |
|  | 모즈습로 | 모습으로 | Appearance | Allative |
|  | 방크법로 | 방법으로 | Way | Allative |
|  | 보더고를 | 보고서를 | Report | Accusative |
|  | 시벰스을 | 시스템을 | System | Accusative |
|  | 실코제는 | 실제로는 | Actually | Topic |
|  | 아둠쉬을 | 아쉬움을 | Regret | Accusative |
|  | 움빔직을 | 움직임을 | Movement | Accusative |
|  | 입케장서 | 입장에서 | Standpoint | Locative |
|  | 인멧터에 | 인터넷에 | Internet | Locative |
|  | 자버전를 | 자전거를 | Bicycle | Accusative |
|  | 주퐁인이 | 주인공이 | The main character | Nominative |
|  | 중베국서 | 중국에서 | China | Locative |
|  | 중프심로 | 중심으로 | Center | Allative |
|  | 처드음로 | 처음으로 | First time | Allative |
|  | 캠빈페을 | 캠페인을 | Campaign | Accusative |
