# Supplementary material for: Neural correlates of confusability in recognition of morphologically complex Korean words
Source: PLoS One. 2021 Apr 15;16(4):e0249111. doi: 10.1371/journal.pone.0249111 (PMC8049294; doi:10.1371/journal.pone.0249111)
Supplement: S2 Table — (DOCX) [file pone.0249111.s003.docx]

S2 Table. Whole brain connectivity table for each seed region.

| **Seed** | **Condition** | **Regions** | **Cluster Size** | **MNI Coordinate (x, y, z)** | **Peak t value** |
| --- | --- | --- | --- | --- | --- |
| **mPFC** | **TSA** | Medial Prefrontal Cortex | 3566 | −2 56 8 | 30.37 |
|  |  | Angular gyrus | 1143 | −48 −64 34 | 6.65 |
|  |  | Superior Temporal gyrus | 103 | 60 −58 20 | 6.31 |
|  |  | Middle Temporal gyrus | 299 | −60 2 −22 | 5.75 |
|  |  | Posterior Cingulum | 208 | −6 −52 28 | 5.24 |
|  |  | Temporal Pole | 66 | 48 22 −28 | 4.94 |
|  |  | Superior Frontal gyrus | 149 | −18 40 40 | 4.85 |
|  | **TSW** | Medial Prefrontal Cortex | 3619 | −2 56 8 | 42.93 |
|  |  | Angular gyrus | 203 | 52 −64 34 | 5.38 |
|  |  | Angular gyrus | 514 | −54 −56 28 | 5.35 |
|  |  | Middle Temporal gyrus | 87 | 54 −10 −16 | 5.06 |
|  |  | Posterior Cingulum | 142 | 6 −50 28 | 4.97 |
| **MTG** | **TSA** | Middle Temporal gyrus | 1116 | −54 −28 −10 | 31.10 |
|  |  | Inferior Frontal gyrus | 164 | 48 34 −16 | 7.61 |
|  |  | Superior Frontal gyrus | 247 | 30 32 56 | 7.11 |
|  |  | Inferior Frontal gyrus | 250 | −48 40 −16 | 7.01 |
|  |  | Superior Parietal lobe | 765 | −36 −68 52 | 6.37 |
|  |  | Middle Frontal gyrus | 249 | −32 16 62 | 6.29 |
|  |  | Middle Temporal gyrus | 494 | 60 −38 −4 | 5.84 |
|  |  | Insular gyrus | 61 | 30 20 −16 | 5.7 |
|  |  | Superior Frontal gyrus | 382 | −14 46 34 | 5.28 |
|  |  | Angular gyrus | 101 | 46 −64 46 | 5.21 |
|  |  | Posterior Cingulum | 52 | 0 −32 32 | 4.9 |
|  | **TSW** | Middle Temporal gyrus | 1072 | −50 −28 −10 | 39.59 |
|  |  | Angular gyrus | 1832 | −38 −68 46 | 8.5 |
|  |  | Inferior Parietal lobe | 256 | 58 −58 38 | 6.17 |
|  |  | Superior Temporal gyrus | 83 | 60 −58 20 | 6.03 |
|  |  | Superior Medial Frontal gyrus | 100 | −8 34 46 | 5.65 |
|  |  | Superior Frontal gyrus | 71 | −12 58 34 | 5.55 |
|  |  | Middle Temporal gyrus | 123 | −54 8 −28 | 5.27 |
|  |  | Inferior Frontal gyrus | 70 | −48 40 −20 | 4.85 |
|  |  | Superior Frontal gyrus | 76 | −14 62 20 | 4.83 |
|  |  | Middle Frontal gyrus | 75 | −32 26 52 | 4.74 |
|  |  | Middle Temporal gyrus | 172 | 70 −26 −10 | 4.72 |
|  |  | Middle Temporal gyrus | 81 | 70 −40 −8 | 4.55 |
| **IPL** | **TSA** | Inferior Parietal lobe | 5347 | −48 −56 46 | 37.79 |
|  |  | Inferior Parietal lobe | 2168 | 46 −56 50 | 11.41 |
|  |  | Middle Frontal gyrus | 3051 | −36 26 44 | 10.46 |
|  |  | Middle Temporal gyrus | 764 | −56 −40 −10 | 7.12 |
|  |  | Cerebellum | 119 | 34 −64 −32 | 6.12 |
|  |  | Middle Temporal gyrus | 423 | 64 −34 −10 | 5.95 |
|  |  | Middle Frontal gyrus | 232 | 36 28 46 | 5.93 |
|  |  | Cerebellum | 199 | −38 −62 −44 | 5.63 |
|  |  | Cerebellum | 156 | 34 −68 −44 | 5.36 |
|  |  | Superior Medial Frontal gyrus | 96 | 4 64 4 | 4.73 |
|  |  | Inferior Frontal gyrus | 112 | −42 38 −16 | 4.6 |
|  | **TSW** | Inferior Parietal lobe | 7538 | −48 −56 46 | 34.27 |
|  |  | Middle Frontal gyrus | 1003 | −36 28 44 | 7.17 |
|  |  | Anterior Cingulum | 636 | −2 40 26 | 7.11 |
|  |  | Middle Frontal gyrus | 517 | −38 52 −2 | 6.69 |
|  |  | Middle Temporal gyrus | 473 | −62 −26 −16 | 6.22 |
|  |  | Middle Temporal gyrus | 375 | 70 −26 −10 | 5.56 |
|  |  | Middle Frontal gyrus | 153 | 42 20 50 | 5.5 |
|  |  | Cerebellum | 56 | 18 −88 −38 | 5.5 |
|  |  | Superior Frontal gyrus | 54 | −18 52 26 | 5.36 |
|  |  | Cuneus | 101 | 16 −64 34 | 5.32 |
|  |  | Middle Frontal gyrus | 91 | 42 38 28 | 5.26 |
|  |  | Precuneus | 125 | −2 −68 34 | 4.98 |
| **IPS** | **TSA** | Intra-Parietal Sulcus  Superior Occipital gyrus  Middle Occipital gyrus | 5976 | −24 −64 34  −20 −68 34  −24 −64 42 | 50.21  19.44  12.93 |
|  |  | Inferior Frontal gyrus  Precentral gyrus  Middle Frontal gyrus | 422 | −38 8 22  −44 4 46  −48 8 44 | 8.39  5.47  4.77 |
|  |  | Inferior Temporal gyrus | 122 | 54 −56 −10 | 6.71 |
|  |  | Supplementary Motor | 238 | 0 16 46 | 6.24 |
|  |  | Inferior Temporal gyrus | 305 | −50 −56 −14 | 5.91 |
|  |  | Inferior Frontal gyrus  Middle Frontal gyrus | 559 | 46 8 22  52 20 40 | 5.88  5.66 |
|  | **TSW** | Intra-Parietal Sulcus  Superior Occipital gyrus  Superior Occipital gyrus | 8254 | −24 −64 34  −20 −68 34  28 −68 34 | 50.42  21.33  11.08 |
|  |  | Inferior Temporal gyrus | 187 | 54 −58 −14 | 6.93 |
|  |  | Inferior Temporal gyrus | 578 | −50 −52 −14 | 6.7 |
|  |  | Inferior Frontal gyrus | 316 | −48 8 20 | 6.25 |
|  |  | Middle Frontal gyrus  Superior Frontal gyrus | 77 | −26 2 68  −24 2 72 | 5.63  4.23 |
|  |  | Inferior Frontal gyrus | 253 | 40 14 20 | 5.48 |
|  |  | Middle Cingulum gyrus  Superior Medial Gyrus  Superior Medial gyrus | 224 | 6 26 40  10 26 44  2 30 38 | 5.33  4.28  4.19 |
|  |  | Supra Marginal gyrus  Inferior Parietal lobe | 77 | 66 −16 28  66 −14 22 | 5.27  4.73 |
| The regions reported were *p* < .05 FDR-corrected at a cluster level, *p* < .001, *K*s > 30 at a voxel level. | | | | | |
